# Pavlovian Extinction and Recovery Effects in Aversive Pavlovian to Instrumental Transfer

**DOI:** 10.3389/fnbeh.2017.00179

**Published:** 2017-09-25

**Authors:** Vincent D. Campese, Ian T. Kim, Gerardo Rojas, Joseph E. LeDoux

**Affiliations:** ^1^Center for Neural Science, New York University New York, NY, United States; ^2^Department of Neuroscience, Carthage College Kenosha, WI, United States; ^3^Emotional Brain Institute, Nathan Kline Institute for Psychiatric Research Orangeburg, NY, United States

**Keywords:** Pavlovian, instrumental, transfer, avoidance, shuttling, rat, extinction

## Abstract

Three studies explored the sensitivity of aversive Pavlovian to instrumental transfer (PIT) to Pavlovian extinction in rodents. Rats underwent Pavlovian conditioning prior to avoidance training. The PIT test then involved assessment of the effects of the Pavlovian conditioned stimulus (CS) on the performance of the avoidance response (AR). Conducting extinction prior to avoidance training and transfer testing, allowed spontaneous recovery and shock reinstatement of extinguished motivation, whereas conducting extinction following avoidance training and just prior to PIT testing successfully reduced transfer effects. This was also the case in a design that compared responding to an extinguished CS against a non-extinguished CS rather than comparing extinguished and non-extinguished groups to one another. While extinction treatments in many appetitive PIT studies do not successfully reduce transfer, and can sometimes enhance the effect, the current findings show that an extinction treatment temporally close to transfer testing can reduce the motivational impact of the aversive Pavlovian CS on instrumental avoidance responding.

## Introduction

Studies of aversive Pavlovian conditioning demonstrate control over species-specific defensive responses (SSDRs: e.g., freezing) by a previously neutral conditioned stimulus (CS) that has been paired with a shock unconditioned stimulus (US; Pavlov, 1927; Blanchard and Blanchard, 1972; Bolles and Fanselow, 1980). Much research has outlined the neural basis of this form of learning (Johansen et al., 2011) and studies into the role of the CS in controlling SSDRs continue today. However, an aversive CS is also capable of controlling the performance of goal-directed behaviors. This is illustrated by aversive Pavlovian-to-instrumental transfer (PIT: Bolles and Popp, 1964; Rescorla and Lolordo, 1965; Rescorla, 1968; Weisman and Litner, 1969; Overmier and Payne, 1971; Overmier and Brackbill, 1977; Patterson and Overmier, 1981) and other related aversive instrumental learning phenomena (see LeDoux et al., 2016). In PIT, a previously trained aversive CS enhances the rate at which subjects perform avoidance behaviors (e.g., two-way shuttling) that terminate threats and prevent harm. Thus, whereas an aversive CS normally produces freezing, if presented while performing a separately trained avoidance response (AR) the CS produces very little freezing and instead facilitates avoidance (see Campese et al., 2013).

Studies of appetitive motivation have extensively examined PIT, and its sensitivity to treatments such as extinction (Delamater, 1996; Holmes et al., 2010; Lovibond et al., 2015; Cartoni et al., 2016) and outcome devaluation (Holland, 2004; Corbit and Janak, 2010). Such analyses have not been performed on aversive PIT. Therefore, in order to better understand the motivational nature of aversive transfer, the experiments reported below explored the sensitivity of aversive PIT to Pavlovian extinction. In Experiment 1, extinction of the CS was conducted prior to avoidance training while in Experiment 2 this treatment was conducted following avoidance. In both experiments the effects of recovery from extinction (e.g., spontaneous recovery and reinstatement) were tested. To determine the specificity of the underlying motivational processes, the effect of extinction was evaluated relative to a non-extinguished CS, rather than to a group that had not undergone extinction. The findings from these studies suggest that aversive PIT is sensitive to sensory-specific extinction processes, including those related to the recovery of extinction. This differs from findings in appetitive PIT where sensitivity to extinction depends more on training duration and testing conditions (Holmes et al., 2010; Laurent et al., 2016).

### Materials and Methods

#### Subjects

One-hundred and eighty-two male Sprague-Dawley rats, purchased from Hilltop Lab Animals (Scottsdale, PA, USA) were the subjects of the studies reported herein. All weighed approximately 275 g at the start of the experimentation. Subjects were housed in standard Plexiglas cages with free food and water available on paper bedding in a colony running a 12:12 light:dark schedule. Animal care and housing was in accordance with the Institutional Animal Care and Usage Committee (IACUC) policies and met the current standards of the Association for Assessment and Accreditation of Laboratory Animal Care International (AAALAC). This study was carried out in accordance with the recommendations of the Guide to the Care and Use of Laboratory Animals, of the National Institutes of Mental Health. The protocol was approved by the New York University Animal Welfare Committee.

#### Apparatus

Aversive Pavlovian conditioning was conducted in a set of four standard conditioning chambers, manufactured by Coulbourn instruments (model no H10-11R-TC; Whitehall, PA, USA). The chambers were housed in sound and light attenuating cubicles and were equipped with a 5 ohm speaker connected to a programmable audio generator (Coulbourn, model A12-33) which delivered the 5 kHz tone and white noise stimuli. This system was capable of delivering shock through stainless steel grid floors using precision animal shockers also manufactured by Coulbourn (model no. H13-15). Each chamber also included a klaxon horn (Wolo, model no. 330; 114 dB), which served as an alternative aversive US in some studies reported below. Behavioral sessions were controlled using Graphic State 3 (by Acimetrics and Coulbourn).

Instrumental avoidance and transfer tests were conducted in two-way shuttle chambers manufactured by Coulbourn instruments (model no H10-11R-SC) and were also in sound and light attenuating shells. These chambers were equipped with the same modules as the Pavlovian chambers described above except for the klaxons. In Experiment 2, flat plastic floors and checkered patterning was used to create a distinct context from the standard avoidance chamber.

## Experiment 1: The Effect of Extinction Treatment Prior to Avoidance Training on Transfer of Motivational Control

**Figure 1:**
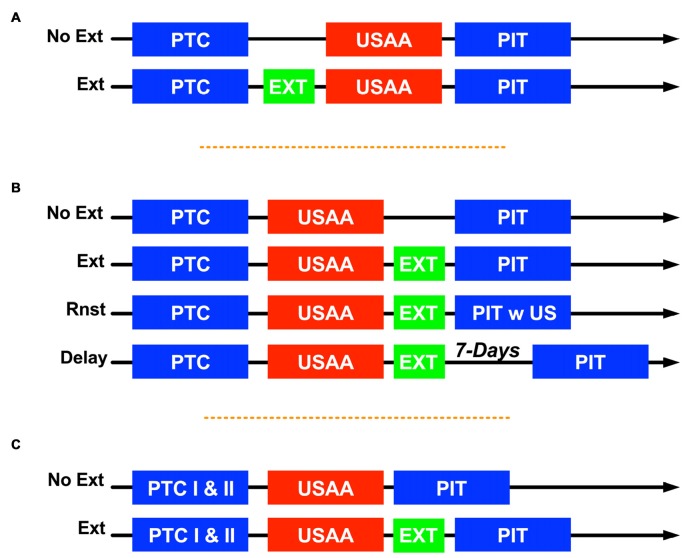
Summary of designs where Pavlovian conditioning (PTC) was followed by unsignaled active avoidance (USAA) and then tests for Pavlovian-to-instrumental transfer (PIT) with extinction (EXT) intervening in various ways for the different groups. Experiment 1 is shown in **(A)**, No Ext subjects were placed in the chamber but did not receive any conditioned stimulus (CS) presentations while EXT subjects received non-reinforced tones between PTC and USAA. For Experiment 2 **(B)** subjects in the Delay group had 1 week between extinction and PIT tests, while Rnst subjects were tested the day after extinction but experienced 2 unsignaled shocks at the start of these sessions. For Experiment 3 **(C)** subjects had two PTC sessions. One session was with shock and one with the klaxon as the unconditioned stimulus (US). Subjects in group No Ext did not receive extinction treatment and PIT was evaluated using a within-subjects approach. The Ext subjects received either tone or noise extinction and then were tested with the tone only.

Typically in studies of PIT, Pavlovian conditioning precedes instrumental training and is followed by transfer testing (Holmes et al., 2010; Cartoni et al., 2016). This is the progression for the aversive PIT task used in the studies below with minor variations where extinction training systematically intervenes between these phases (Campese et al., 2014; see Figure 1 below). While the underlying factors that enhance avoidance responding during aversive transfer are unknown, one possibility may depend on generalized motivation from the avoidance context to the CS. Such generalization could be mediated by footshock exposure during the Pavlovian and avoidance training phases (Honey and Hall, 1989). Because the tone CS and the avoidance context are both paired with the shock US early in training they may acquire equivalence over the course of training, increasing generalization between these cues. If this were the case, extinction of the CS might prevent PIT or reduce avoidance more generally via this mechanism. Therefore, in Experiment 1, extinction was conducted in the non-avoidance context prior to avoidance training to determine whether this might prevent or reduce acquired generalization and eliminate transfer. Control subjects were exposed to the chamber during extinction, but the CS was not presented.

### Pavlovian Conditioning

Pavlovian conditioning was conducted in a single session in a standard Pavlovian chamber. Following a 5-min baseline, there were three trials in which the 30-s tone CS co-terminates with a 1-s 0.7 mA footshock US. Trials were separated by a 180-s intertrial interval (ITI) and the session duration was 15-min.

### Extinction

Subjects received a single session of extinction in the Pavlovian chamber the day before unsignaled active avoidance (USAA) training began. Following a 3-min baseline, 25 1-min duration CS trials occurred and were separated by a 15 s ITI. The session ended 2 min following the end of last trial. The total session duration was 33 min. Control subjects were exposed to the chamber but did not receive any extinction trials with the CS.

### Unsignaled Sidman Active Avoidance (USAA)

USAA took place in the two-way shuttle chambers over 15 sessions. Typically, there were four or five sessions per week, with weekends off, and with one session per day. During the 25-min long sessions, subjects received a 0.5-s duration footshock (also 0.7 mA) every 5 s (i.e., a shock-shock or *S-S* interval). In order to establish a steady rate of unsignaled shuttling, each shuttle response was reinforced by 30 s of shock free time (i.e., a response-shock or *R-S* interval of 30 s). Additionally upon completion of a shuttle response, a feedback cue was presented in the form of a 0.3-s blinking of the overhead houselights. Subjects that did not meet inclusion criteria during this phase (two consecutive sessions with more than 20 ARs; see Lázaro-Muñoz et al., 2010) were excluded following day 10. Following day 15 of USAA training subjects that met training criteria move to the next phase.

### Pavlovian-to-Instrumental Transfer (PIT) Testing

PIT testing was conducted as previously reported in the avoidance chambers (Campese et al., 2013, 2014). There were two test sessions with one on each of two consecutive days (i.e., separated by 24 h). Each session included a single CS presentation used to measure PIT. In these sessions, subjects shuttled under extinction for the first 15-min of the test (i.e., no shocks are presented). At this point, the software controlling the experiment then began to monitor response rates and presented the CS to each subject independently when the response rate for that subject dropped to two responses per minute for a full 2 min (see Campese et al., 2013). The CS then remained on until 10 responses were made, at which point the session ended and house lights turned off. The blinking-light feedback cue was still presented following shuttle-responses during the test phase. Following the end of the session, subjects were returned to the colony and given a second identical test the following day.

### Results

**Figure 2:**
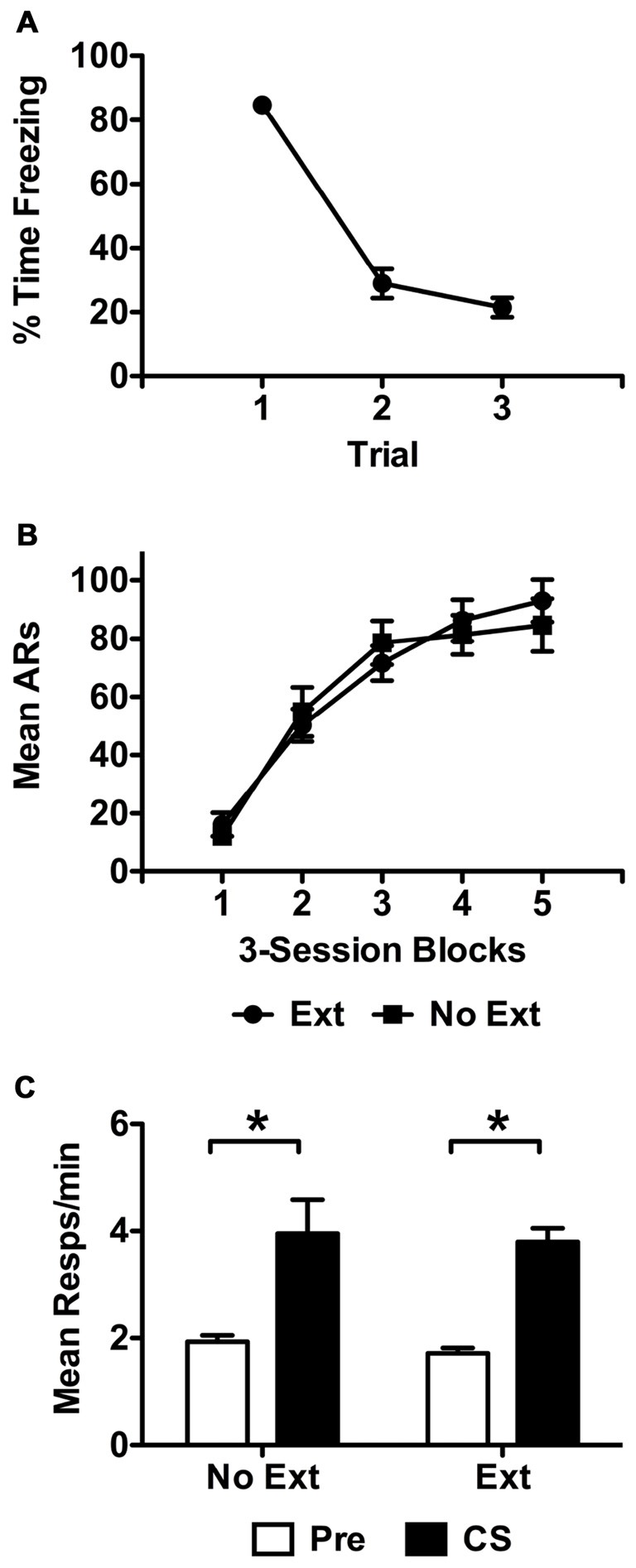
Extinction data for subjects in Experiment 1 are presented in **(A)** as mean percent time freezing over the 60 s trials for subjects exposed to the tone during this phase. Control subjects did not receive stimulus presentations during this phase. Representative trials 1, 13 and 25 are presented from this phase for extinguished subjects only. Acquisition data from the USAA phase are presented in **(B)** as mean avoidance responses (ARs) per 3-session block of training. Transfer test data are presented in f **(C)** in terms of responses per minute during the Pre and CS recording intervals for each group. Asterisks indicate statistical significance at alpha = 0.05.

Extinction data are presented in Figure 2A in terms of mean percent time freezing to the CS over representative trials. These data were analyzed using a repeated measure analysis of variance (ANOVA) with trial as the within-subjects factor. This analysis found that freezing to the CS reduced over the course of the extinction session, *F*_(2,32)_ = 173.37, *p* < 0.001. Data from the USAA phase are presented in Figure 2B in terms of mean shuttle responses per 3-session block of training. Final sample sizes were 16 and 10 subjects for groups *Ext* and *No Ext* respectively. These data were analyzed with a split-plot repeated measures ANOVA including *Group* (Ext vs. No Ext) as the between-subjects factor and Training *Block* as the within-subjects factor. This analysis found that responding increased over training blocks (effect of *Block*: *F*_(4,96)_ = 70.55, *p* < 0.001) comparably for both groups (*Block* × *Group*: *F*_(4,96)_ = 0.816, *p* = 0.52; effect of *Group*: *F*_(1,24)_ = 0.024, *p* = 0.88).

Data from the transfer test phase are presented in Figure 2C in terms of responses per minute during the pre and CS intervals. These data were analyzed using a split-plot repeated measures ANOVA with *Interval* (Pre vs. CS) as the within-subjects factor and *Group* (No Ext vs. Ext) as the between-subjects factor. Because preliminary analyses found no differences between tests 1 and 2 (effect of *Test*: *F*_(1,24)_ = 2.79, *p* = 0.11) or interactions between test and other factors (*Test* × *Group*: *F*_(1,24)_ = 0.17, *p* = 0.68; *Test* × *Interval*: *F*_(1,24)_ = 1.36, *p* = 0.26; *Test* × *Interval* × *Group*; *F*_(1,24)_ = 0.06, *p* = 0.82) data were collapsed across test and are presented as an average for both groups. Analysis of these data found that subjects in both groups showed comparable facilitation of responding by the shock-paired CS, (effect of *Interval*: *F*_(1,24)_ = 51.76, *p* < 0.001; effect of *Group*: *F*_(1,24)_ = 0.43, *p* = 0.52; *Interval* × *Group*: *F*_(1,24)_ = 0.01, *p* = 0.93).

### Discussion

The results from Experiment 1 show intact facilitation of avoidance responding by a shock-paired CS following an extinction treatment that occurred prior to avoidance training. While these subjects performed comparably to non-extinguished controls during the transfer test, extinction clearly reduced conditioned freezing responses during the extinction phase, suggesting the treatment had been successful. However, the duration and nature of the USAA experience leaves open the possibility that extinction recovery phenomena, such as reinstatement and spontaneous recovery were engaged, causing a return of motivation relevant to PIT performance. The USAA phase duration was 15 training days, and these sessions involved many unsignaled footshock USs. These treatments could have easily engaged spontaneous recovery and reinstatement mechanisms. Furthermore, PIT testing takes place outside of the extinction context, allowing renewal processes to potentially drive recovery as well. The possible contribution of spontaneous recovery and reinstatement effects to PIT following extinction was examined in Experiment 2 below.

## Experiment 2: The Effect of Extinction Treatment Following Avoidance Training on Transfer of Motivational Control

In order to determine whether extinction can influence aversive transfer with minimized impact of recovery phenomena, extinction was conducted the day following the end of USAA (i.e., the day before PIT testing). In addition to the main Extinction and No-Extinction groups, other conditions were included to determine whether spontaneous recovery (Delay) or reinstatement (Rnst) treatments promoted recovery of PIT following extinction in Experiment 1. While the former condition simply had a delay interposed between extinction and PIT testing, the latter group was exposed to inescapable and unsignaled footshock USs at the start of the test sessions. Previous studies have shown that the effects of time and context are additive in renewal or context-based recovery effects (Bouton et al., 2006). Thus, if extinction were capable of reducing transfer, both reinstatement and the passage of time could be expected to result in moderate recovery relative to extinguished subjects and non-extinguished controls. However, if recovery were more dependent upon an additive or interactive process, these treatments alone might be insufficient and these groups would be expected to show no CS-based enhancement of avoidance behavior.

### Pavlovian Conditioning and USAA

These phases were conducted as described above. There was no intervening extinction session between these phases in Experiment 2.

### Extinction

Subjects in Experiment 2 received extinction the day following the end of USAA training. Aside from when the sessions occurred, extinction was conducted as described above, along with the use of non-extinguished controls that were only exposed to the chamber (see Figure 1B).

### Pavlovian-to-Instrumental Transfer (PIT) Testing

Subjects in Experiment 2 underwent transfer testing the day after extinction, except for the spontaneous recovery group, for which there was a 7-day break that was spent in the home cages before the test day. The PIT tests were conducted in the same manner as Experiment 1, except for the reinstatement group. For these subjects, the test sessions began with two unsignaled inescapable 1-s duration 0.7 mA footshocks separated by 10 s.

### Results

**Figure 3:**
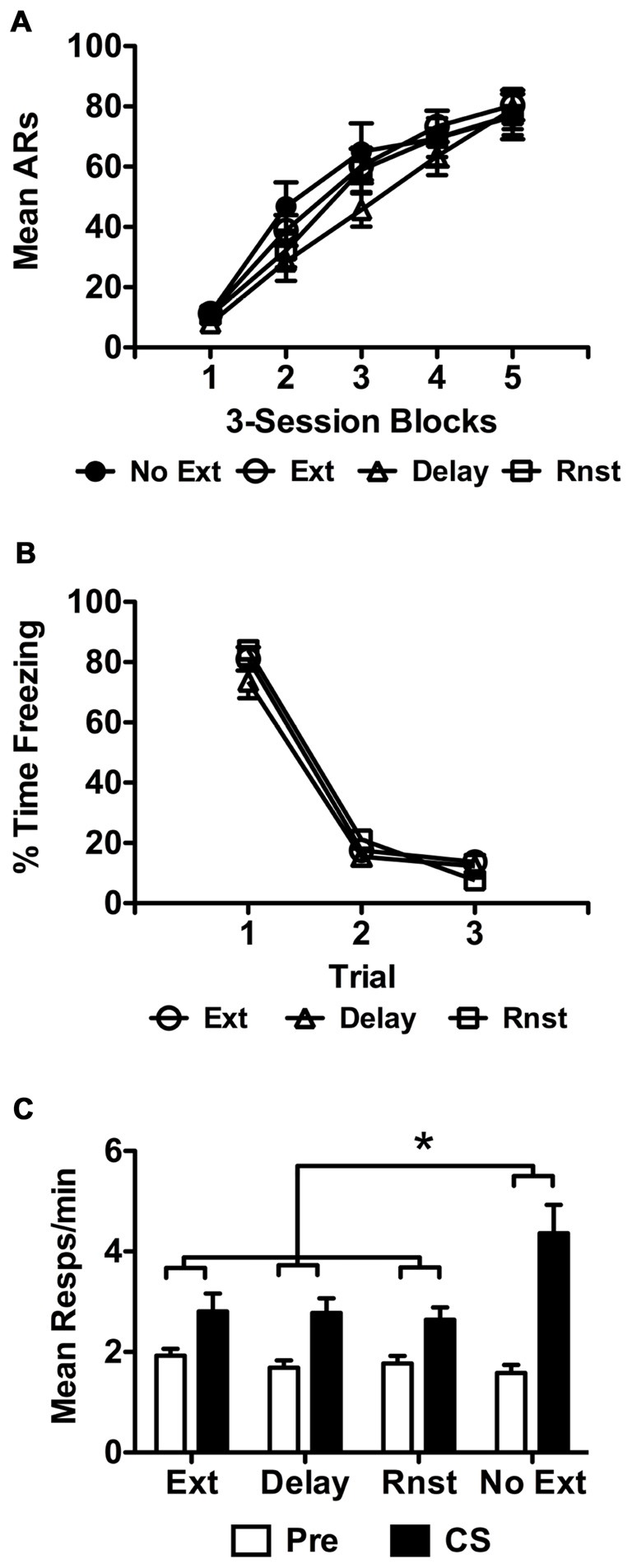
**(A)** depicts USAA acquisition for all groups, including non-extinguished subjects. **(B)** shows extinction only for the groups exposed to the tone CS during this phase of Experiment 2 as previously described. Transfer test data from Experiment 2 are presented in terms of Pre and CS responses as previously described in **(C)** for each group in the study. Asterisks denote statistical significance at alpha = 0.05.

Data from the USAA phase are presented below in Figure 3A for each group as previously described. The final sample size was 11 for non-extinguished control subjects. Of the extinguished rats, 16 were assigned to the reinstatement (Rnst) and 15 to the spontaneous recovery (Delay) conditions, with the remaining 14 tested the day following extinction as previously described. These data were analyzed using a split-plot repeated measures ANOVA with *Block* as the within-subjects factor and *Group* (No Ext, Ext, Rnst or Delay) as the between-subjects factor. This analysis revealed a significant effect of *Block*, *F*_(4,212)_ = 146.86, *p* < 0.001, indicating that responding increased over training. The effect of *Group* was not significant, *F*_(3,53)_ = 0.65, *p* = 0.59, and neither was the *Group* × *Block* interaction, *F*_(12,212)_ = 1.07, *p* = 0.39.

Freezing data during extinction for each group exposed to the CS are presented in Figure 3B as previously described. Due to a hard drive error extinction data for three subjects from the Rnst and Delay groups were not retrievable. The data for the rest of the subjects were analyzed with a split-plot repeated measures ANOVA, which found that freezing reduced over trials during this phase, *F*_(2,72)_ = 355.09, *p* < 0.001, and did so comparably for each group, *F*_(4,72)_ = 1.33, *p* = 0.27. This included no overall effect of *Group*, *F*_(2,36)_ = 1.60, *p* = 0.22.

Data from the transfer tests are presented below in Figure 3C for each group as described earlier and were analyzed using the same approach. Data were collapsed across tests since preliminary analyses including *Test* as an additional factor found no main effect (*F*_(1,52)_ = 1.02, *p* = 0.32) or interaction with this factor at the source (*Test* × *Interval*: *F*_(1,52)_ = 0.01, *p* = 0.94; *Test* × *Interval* × *Group*: *F*_(3,52)_ = 0.15, *p* = 0.93). Analysis of the collapsed data revealed a significant effect of *Interval*, *F*_(1,52)_ = 49.28, *p* < 0.001, *Group*, *F*_(3,52)_ = 3.29, *p* = 0.028 and a significant *Interval* × *Group* interaction, *F*_(3,52)_ = 4.58, *p* = 0.006. Bonferroni corrected *post hoc* tests found more overall responding in non-extinguished (No Ext) controls relative to reinstatement (Rnst) treated subjects (*p* = 0.038). No other *Group* comparisons were significant. Follow up pairwise comparisons to identify the source of the *Interval* × *Group* interaction found that this effect was due to higher responding during the CS in the No Ext group compared to all other groups (No Ext — *M* = 4.37, 95% CI [3.26 5.48]; Ext — *M* = 2.81, 95% CI [2.10 3.52]; Delay — *M* = 2.79, 95% CI [2.23 3.35]; Rnst — *M* = 2.64, 95% CI [2.17 3.12]).

### Discussion

The results of Experiment 2 show normal transfer in control subjects that did not receive extinction treatment (Campese et al., 2013). Successful extinction was seen in subjects given exposure to the CS in all cases and conducting extinction just prior to PIT testing (rather than before avoidance) attenuated the transfer effect. Baseline shuttling was comparable in extinguished and non-extinguished subjects suggesting avoidance behavior in general was not influenced by extinction. Rather the effect of Pavlovian extinction was a reduction in aversive PIT specifically.

Subjects without any intervening treatments following extinction showed significantly less response elevation by the CS than the non-extinguished control group. The results from the reinstatement and delay groups together with those from Experiment 1 suggest that while the passage of time or exposure to unsignaled footshocks alone have very little impact on recovery, these processes may interact in an additive manner when extinction takes place distally from transfer testing. This could have served to reinstate motivation more strongly in Experiment 1, where subjects experienced footshocks during USAA over the 15 days following extinction. Alternatively, the treatments applied above may have simply been insufficient to engage these recovery mechanisms. However, this is unlikely, as in studies of Pavlovian conditioning 1-week intervals are typically used to promote spontaneous recovery (Rescorla, 2004) and a small number of shocks normally used to reinstate CRs (Bouton, 2004). It should also be pointed out that PIT testing took place outside of the extinction context, which would be expected to cause renewal and prevent expression of extinction. Despite this context switch, subjects showed reduced transfer, although the enhancement effect did persist in an attenuated form. Whether conducting extinction in the USAA context would completely abolish the effect cannot be determined from the current study, but these results clearly show that extinction following USAA reduces aversive transfer.

## Experiment 3: Does Extinction Generalize between Pavlovian Cues in Aversive Pavlovian to Instrumental Transfer?

To determine whether the effect of extinction was specific to the treated CS and not a general reduction of motivational enhancement, a within-subjects aversive PIT design was employed. Subjects received two Pavlovian training sessions at the start of the study. In one session a CS (e.g., tone) was paired with footshock, and in the second session, another CS (e.g., noise) was paired with an aversive horn (i.e., klaxon; see Figure 1). Subjects then underwent USAA and transfer tests. Non-extinguished subjects were first tested to evaluate transfer in this within-subjects design. Then extinguished subjects (i.e., extinction following USAA) were tested after the same training procedure. We expected enhancement of USAA by both shock and klaxon-paired stimuli, and that extinction would produce a selective reduction in the transfer effect.

### Pavlovian Conditioning

Subjects in Experiment 3 underwent two Pavlovian conditioning sessions over 2 days using the parameters previously described. On the first day, CS1 (tone or noise) was paired with US1 (either a 1-s footshock or a 5-s 114 dB klaxon horn) and the next day, CS2 was paired with US2. These sessions were counterbalanced with regards to order and stimulus combinations. It should be noted, that the study was run in two separate replications, which is considered as a factor in the analysis offered below.

### USAA and Extinction

The day following the second Pavlovian conditioning session, subjects moved to the next phases of the study, USAA, and then extinction. These phases were conducted as described above for Experiment 2. The day following the end of USAA subjects received extinction. During extinction, half of the subjects received tone extinction, the other half received noise. These cues were counterbalanced so that for half of each group, the cue was paired with shock, and for the other half, klaxon. All other parameters were the same as described. Non-extinguished subjects were trained as described above but simply began testing the day following the end of USAA and, thus, did not receive an extinction session.

### PIT Testing

#### Non-Extinguished Subjects

The day after the end of USAA, PIT tests began where half of the subjects received the tone CS during tests 1 and 2 while the other half received tests with the noise CS. These assignments were reversed for an additional two tests such that each subject was evaluated for the shock-paired and klaxon-paired CS’s ability to augment USAA in a fully counterbalanced fashion with two tests per cue.

#### Extinguished Subjects

The day following extinction, PIT testing began. PIT testing was conducted over two sessions with tone as previously described and included no other treatments. Counterbalancing rendered the test cue (tone) extinguished for half of the subjects but not the other half, and further balanced these groups on the basis of the outcome signaled by the CS (i.e., shock or klaxon).

### Results

#### Non-Extinguished Subjects

**Figure 4:**
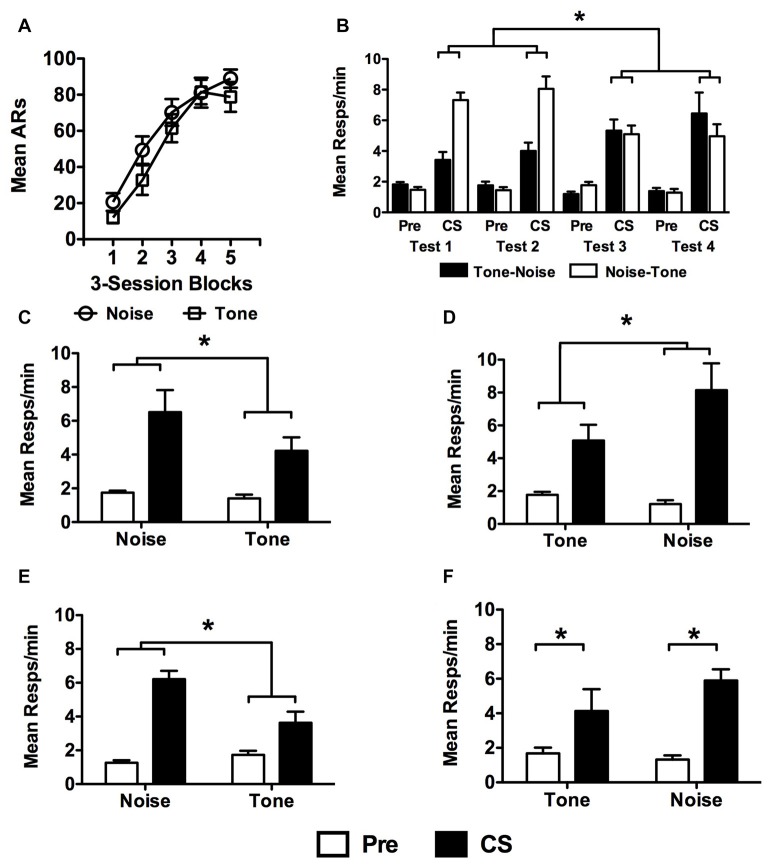
Data from non-extinguished subjects in Experiment 3. USAA training data from are presented in **(A)** as previously described, but as a function of which cue predicted shock during Pavlovian conditioning (i.e., tone or noise). **(B)** Test data are presented for each of the four PIT tests separately for subjects tested first with tone and those tested first with noise. There were two sessions with each stimulus (i.e., tone and noise). PIT Test data were collapsed across test wave (i.e., tests 1 and 2 vs. tests 3 and 4) and are presented for subsets of the Noise-Shock (**C**: Noise-Tone and **D**: Tone-Noise) and Tone-Shock (**E**: Noise-Tone and **F**: Tone-Noise) conditions separately to show the test sequence effects. Asterisks signify statistical significance at the alpha = 0.05 level.

USAA acquisition data are presented in Figure 4A in terms of the stimulus predicting shock (i.e., Tone vs. Noise) for non-extinguished subjects. These data were analyzed with a *Block* (1–5) × *Group* (Tone vs. Noise CS-shock counterbalancing condition) split-plot repeated measures ANOVA which found a significant effect of *Block*, *F*_(4,72)_ = 59.28, *p* < 0.001, no main effect of *Group*, *F*_(1,18)_ = 1.39, *p* = 0.25, or an interaction between these factors, *F*_(4,72)_ = 0.67, *p* = 0.62.

Shuttling data from the PIT test phase are presented in Figure 4B in terms of responses per minute during the Pre CS and CS periods. Data are presented for each test separately for Tone-Noise (*N* = 9) and Noise-Tone (*N* = 11) tested subjects. These data were analyzed with a split-plot repeated measures ANOVA comparing the within-subjects factors of *Test* (1–4) and *Interval* (Pre vs. CS) with the between-subjects factors of *CS-Shock* (Tone vs. Noise) and *CS-Test Order* (Tone-Noise vs. Noise-Tone). The only significant main effect revealed was for Interval, *F*_(1,16)_ = 93.16, *p* < 0.001. The effects of *Test*, *F*_(3,48)_ = 0.59, *p* = 0.63, *CS-Shock*, *F*_(1,16)_ = 0.32, *p* = 0.58 and *CS-Test Order* were not significant, *F*_(1,16)_ = 1.88, *p* = 0.19. While most of the interaction tests did not produce significant results (*Test* × *CS-Shock*, *F*_(3,48)_ = 0.06, *p* = 0.98; *Test* × *CS-Shock* × *CS-Test Order*, *F*_(3,48)_ = 2.38, *p* = 0.08; *Interval* × *CS-Test Order*, *F*_(1,16)_ = 2.43, *p* = 0.14; *Interval* × *CS-Shock*, *F*_(1,16)_ = 0.16, *p* = 0.70; *Interval* × *CS-Shock* × *CS-Test Order*, *F*_(1,16)_ = 0.39, *p* = 0.54; *Test* × *Interval*, *F*_(3,48)_ = 0.80, *p* = 0.50; *Test* × *Interval* × *CS-Shock*, *F*_(3,48)_ = 0.73, *p* = 0.54; *Test* × *Interval* × *CS-Test Order*, *F*_(3,48)_ = 2.02, *p* = 0.12; *CS-Test Order* × *CS-Shock*, *F*_(1,16)_ = 0.53, *p* = 0.48), the following effects were found: significant interactions were revealed between the *Test* × *CS-Test Order* factors, *F*_(3,48)_ = 11.05, *p* < 0.001 as well as the three factor comparison of the *Test* × *Interval* × *CS-Test Order* effect, *F*_(3,48)_ = 13.05, *p* < 0.001. Bonferroni corrected comparisons found that these effects reflected more responding to noise than tone during tests 1, 2 and 4 (Test 1 – Tone, *M* = 3.1, 95% CI [1.91 4.31]; Noise, *M* = 6.9, 95% CI [5.85 8.02]); Test 2 – Tone, *M* = 3.6, 95% CI [2.03 5.13]; Noise, *M* = 7.7, 95% CI [6.30 9.10]); Test 4 – Tone, *M* = 4.5, 95% CI [2.22 6.87]; Noise, *M* = 7.1, 95% CI [4.48 9.63]).

To highlight these effects, data are also presented collapsed over tests 1 and 2, as well as 3 and 4 separately for *CS-Shock* (Noise-Shock in Figures 4C,D and Tone-Shock in Figures 4E,F) and by *CS-Test Order* (Noise-Tone or Tone-Noise testing sequences). An additional analysis on these data compliment the overall analysis by showing that while more responding was generally seen to the noise CS, PIT was observed for each CS in each subgroup. Noise-Shock trained subjects showed more responding to noise when tested with noise first (Noise, *M* = 6.52, 95% CI [4.30 8.73]; Tone, *M* = 4.22, 95% CI [2.21 6.24]; Figure 4C) or tone first (Tone, *M* = 5.08, 95% CI [2.66 7.51]; Noise, *M* = 8.14, 95% CI [5.93 10.35]; Figure 4D). Tone-Shock trained subjects tested with noise first also showed this difference (Noise, *M* = 6.21, 95% CI [3.50 8.92]; Tone, *M* = 3.63, 95% CI [1.16 6.09]; Figure 4E). For Tone-Shock training subjects first tested with tone, this was not significant but the means showed a similar pattern (Tone, *M* = 4.13, 95% CI [1.71 6.56]; Noise, *M* = 5.90, 95% CI [3.69 8.10]; Figure 4F).

#### Extinguished Subjects

**Figure 5:**
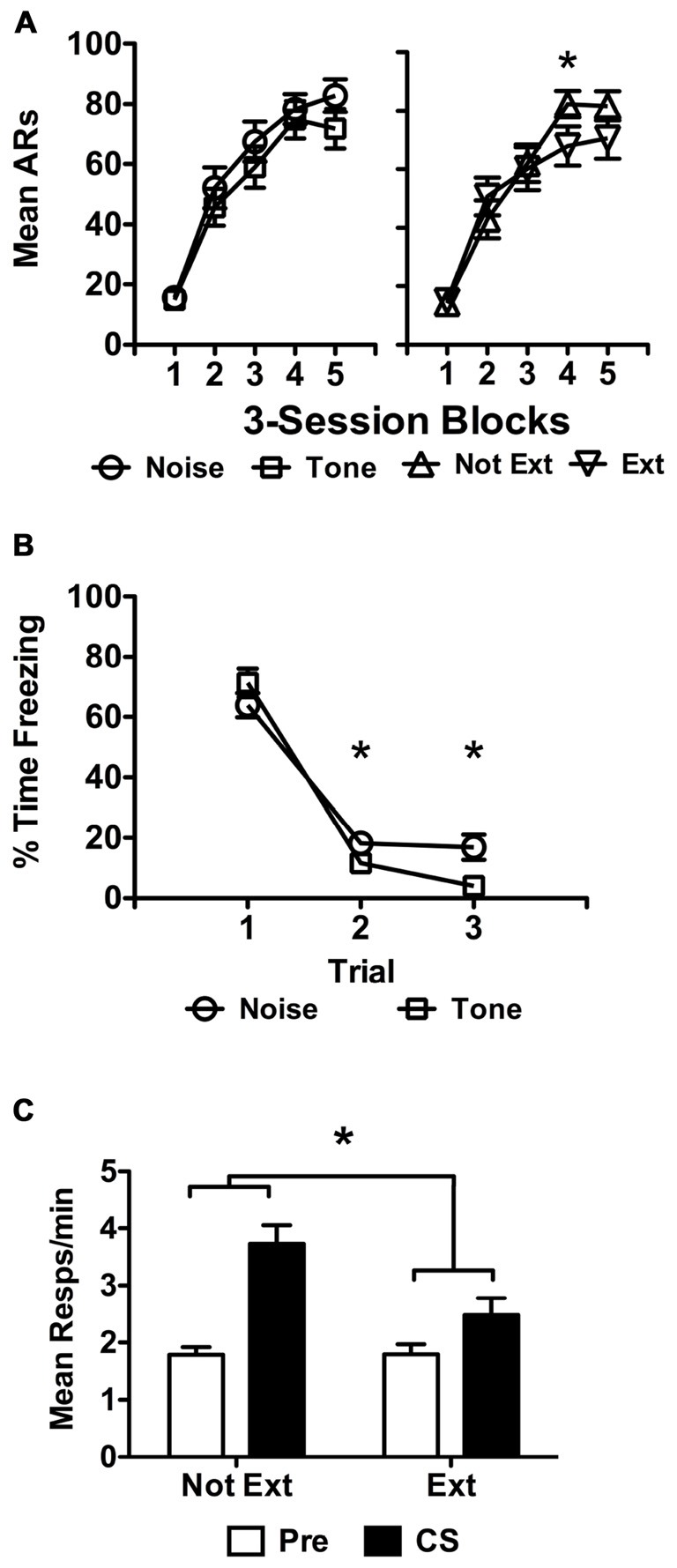
USAA training data are presented in **(A)** as previously described for extinguished subjects in Experiment 3. Extinction data for the subjects treated with tone or noise are presented separately in **(B)** as done above. Transfer test data are presented in panel **(C)** in terms of responses per minute as previously described. Statistical significance at alpha = 0.05 is signified by asterisks.

Acquisition data from the USAA phase are presented in Figure 5A (left panel) as a function of counterbalancing condition with regards to the Pavlovian cue that predicted shock. These data were analyzed using a *Block* (1–5) × *Group* (Tone vs. Noise) split-plot repeated measures ANOVA. Responding significantly increased over three-session blocks, *F*_(4,160)_ = 101.6, *p* < 0.001. This was true for both groups, *F*_(1,40)_ = 1.64, *p* = 0.21, with no significant interactive effects, *F*_(4,160)_ = 0.79, *p* = 0.53. These data are also presented in terms of later assignment during the extinction and test phase in Figure 5A (right panel). Using the same analytic procedure on these data similarly reveals a significant effect of *Block*, *F*_(4,160)_ = 107.64, *p* < 0.001, and no main effect of *Group*, *F*_(1,40)_ = 0.30, *p* = 0.59. Generally, responding increased over blocks (*p* < 0.001), except for between blocks 4 and 5, where no difference was found (*p* = 1.0). Additionally, in this analysis a significant *Block* × *Group* interaction was observed, *F*_(4,160)_ = 3.38, *p* = 0.011. Pairwise comparisons showed that in Block 4 responding was higher for subjects that were eventually tested with the non-extinguished CS (M_Not Ext_ = 82.28, 95% CI [70.78 93.78]; M_Ext_ = 67.87, 95% CI [56.37 79.37]). Importantly, no other differences were found amongst the blocks between the groups.

Freezing data from the extinction phase are presented below in Figure 5B for the different stimuli extinguished, tone and noise. Of the 42 subjects remaining following exclusions for poor performance, half received extinction with tone and the other half with noise. Because all subjects were later tested with tone, subjects extinguished with tone became the Ext group, while those extinguished with noise became the Not Ext group, with group names referring to the status of the tone. The data were analyzed with a split-plot repeated measures ANOVA including *Block* (1 vs. 2 vs. 3) as a within-subjects factor and *Extinction* (Ext vs. Not Ext) as a between-subjects factor. This analysis found a significant effect of *Block*, *F*_(2,80)_ = 265.17, *p* < 0.001 and a significant *Block* × *Extinction* interaction, *F*_(2,80)_ = 7.05, *p* < 0.01, with no effect of *Extinction*, *F*_(1,40)_ = 1.17, *p* = 0.29. Bonferroni corrected pairwise comparisons of the *Block* × *Extinction* interaction found that the amount of freezing in blocks 2 and 3 was different for the two groups, with more freezing to the noise in both cases (Block 2 — *M*_Noise_ = 11.0, 95% CI [7.64 14.36]; *M*_Tone_ = 7.0, 95% CI [3.85 10.25]; Block 3 — *M*_Noise_ = 10.5, 95% CI [6.67 14.23]; *M*_Tone_ = 2.5, 95% CI [−1.11 6.11]). No other comparisons were significant for the interaction. However, analyses also revealed that each level of the *Block* factor was significantly different, indicating that despite the block 2 and 3 differences, freezing reduced overall in each subsequent block, (Block 1 vs. Block 2, *M* = 31.45, *p* < 0.001; Block 1 vs. Block 3, *M* = 33.99, *p* < 0.001; Block 2 vs. Block 3, *M* = 2.55, *p* = 0.048).

Data from the transfer test phase are presented in Figure 5C. Preliminary analyses found that tests 1 and 2 were significantly different from one another, *F*_(1,40)_ = 13.25, *p* = 0.001, due to less overall responding in test 2 than test 1. While in studies reported above, comparisons were made between Extinction and No Extinction control groups, in the current study all subjects received an extinction treatment. This extinction treatment may have resulted in generalization following test 1 resulting in reduced responding on test 2. Therefore, test 2 data were not included and test 1 data are presented alone to evaluate the effect of extinction on PIT without this possible extraneous influence. These data were analyzed using a split-plot repeated measures ANOVA including *Interval* (Pre vs. CS) as a within-subjects factor against *Replication* (1 vs. 2), *CS-Shock* (Tone vs. Noise), and *Extinction* (Ext vs. Not Ext) as between-subjects factors. This analysis found a significant effect of *Interval*, *F*_(1,32)_ = 16.18, *p* < 0.001 and a significant effect of *Extinction*, *F*_(1,32)_ = 9.04, *p* = 0.005. There were no effects of *Replication*, *F*_(1,32)_ = 0.01, *p* = 0.93 or *CS-Shock*, *F*_(1,32)_ = 0.01, *p* = 0.91, nor any significant interactions between these and the other factors included in the analysis (*Replication* × *Extinction*, *p* = 0.84; *Replication* × *Interval*, *p* = 0.88, *Replication* × *CS-Shock*, *p* = 0.53, *Replication* × *Extinction* × *CS-Shock*, *p* = 0.45, *Replication* × *Interval* × *Extinction*, *p* = 0.65, *Replication* × *Interval* × *CS-Shock*, *p* = 0.79, *Replication* × *Extinction* × *Interval* × *CS-Shock*, *p* = 0.98; *CS-Shock* × *Extinction*, *p* = 0.32; *CS-Shock* × *Interval*, *p* = 0.37, *CS-Shock* × *Interval* × *Extinction*, *p* = 0.23). The data were collapsed across *Replication* and *CS-Shock* and re-analyzed as they are presented in Figure 5C, including *Interval* and *Extinction*, the factors of interest, in a split-plot repeated measures ANOVA. This analysis revealed a significant effect of *Interval*, *F*_(1,38)_ = 19.69, *p* < 0.001, *Extinction*, *F*_(1,38)_ = 11.12, *p* = 0.002 and a significant interaction between these factors, *F*_(1,38)_ = 4.52, *p* = 0.040. Follow up testing found more shuttling during the tone for noise-extinguished than for tone-extinguished subjects (*M*_Not Ext_ = 3.73, 95% CI [3.11 4.35]; *M*_Ext_ = 2.48, 95% CI [1.83 3.13]).

It should be noted that two outliers were eliminated from this analysis due to excessive responding. One subject from each replication was removed from the extinguished condition due to response rates well above the mean for their group (i.e., more than two standard deviations). While inclusion of these subjects does not change the overall impression of the results, the extent of variability introduced compromises the analysis and obscures the interaction effect. With these subjects, the overall extinguished cue group mean for CS responding is skewed to 3 responses per minute, relative to the 3.7 responses per minute mean seen in the non-extinguished cue subjects. Without these subjects the mean is 2.5 responses per minute for the group tested with the extinguished cue. The critical statistical effects are still obtained with *Replication* included as a factor in the analysis.

## Discussion

The findings of this study replicate the aversive PIT effect with a CS for footshock, but do so using a within-subjects design. This procedure extends what is known about the underlying motivational substrates of aversive PIT by showing that footshock avoidance behavior can be modulated by a cue for an aversive event that is perceptually distinct from footshock (i.e., klaxon). In appetitive PIT studies the congruence between the signaled outcome and that related to the instrumental response has revealed different forms of motivation based on either sensory-specificity when there is congruence or general motivation when these outcomes are incongruent. In the context of the current findings, this may suggest that the facilitation of footshock avoidance by a shock-paired CS can be understood in terms of sensory-specific processes whereas augmentation of responding by a klaxon-paired CS captures general motivational processes. More work is needed to address these questions, but the procedure used in this study can be an important starting point. The findings of this study also replicate the effects of Pavlovian extinction following USAA training on aversive PIT seen in Experiment 2. All subjects underwent extinction in the current study, but PIT was only significantly reduced when the extinguished cue underwent transfer testing. Tests with the non-extinguished cue resulted in normal transfer effects. These results indicate that the extinction treatment selectively influences motivational processes for the extinguished cue, leaving others intact. If klaxon-based as opposed to shock-based signaling does in fact evoke different motivational control of responding, the selectivity of extinction may only apply at that level. In other words, cues also paired with shock may show reduced transfer if a shock paired cue is extinguished for that subject rather than a klaxon-paired cue. Further studies would be needed to address this possibility.

## General Discussion

The findings of the studies above characterize the effect of Pavlovian extinction treatment on aversive motivation assessed via USAA behavior. Whereas non-extinguished controls showed normal facilitation of USAA by a previously shock-paired tone (see Campese et al., 2013), Pavlovian extinction between avoidance training and PIT testing significantly reduced transfer. This was not the case when extinction was conducted between the Pavlovian and avoidance phases. Furthermore, the effect of extinction at this point in training was specific to a CS that had undergone extinction. No evidence for recovery of extinction was observed in subjects exposed to unsignaled shock at the start of transfer tests or given a long delay between extinction and testing. These findings suggest that the recovery of PIT seen when extinction precedes avoidance training may be due to interactions between reinstatement and spontaneous recovery processes over the course of USAA (Rosas and Bouton, 1998).

It should be noted that context-specific extinction or “renewal” processes did not strongly influence PIT, but may still have had some impact. Testing took place in a different context from extinction in all cases, which would be expected to engage renewal mechanisms (Bouton, 2004). For example, because Pavlovian acquisition and extinction took place in the same physical context with PIT testing conducted in the avoidance chamber, these designs can be interpreted in terms of AAB renewal (Bouton and Ricker, 1994). However, testing the CS outside of the extinction context nevertheless resulted in an attenuation of the effect. Despite the reduction in enhancement, the CS was still weakly capable of augmenting responding above baseline. It is possible that this persistent modulatory effect survived due to the absence of the extinction context. Studies conducting extinction in the avoidance context with the response restricted would be needed to address this possibility.

Findings in appetitive PIT suggest that this factor may be important in determining the expression of extinction. Laurent et al. (2016) observed different effects of the CS on instrumental choice when it was tested inside compared to outside of the extinction context. Testing in the extinction context revealed inhibitory effects where choice was biased away from the response associated with the extinguished CS via the outcome. On the other hand, testing the CS outside of the extinction context produced normal PIT (with selective enhancement by outcome in the choice test), which contrasts with the current findings of attenuated transfer using an aversive design. However, findings of studies examining the effect of extinction on appetitive PIT have been mixed. While some have found no impact of extinction (Delamater, 1996), others have found increased transfer by eliminating competing Pavlovian responses (i.e., magazine approach) in over-trained animals through extinction (see Holmes et al., 2010).

In related studies, Rescorla and Lolordo (1965) and Bull and Overmier (1968) used a signaled avoidance procedure where excitatory and inhibitory stimuli showed bidirectional control of baseline avoidance in dogs. While Laurent et al. (2016) used choice to demonstrate the influence of inhibition, Rescorla and Lolordo (1965) and Bull and Overmier (1968) demonstrate this using a single response aversive design more similar to ours. However, these studies used explicit inhibition training procedures rather than extinction, which may be more capable of inhibiting avoidance than extinction. Because the task used in the current studies was designed to isolate facilitation by the CS, it may require parametric changes to detect inhibitory effects of the CS. Nevertheless, extinction clearly produced a reduction in aversive transfer regardless of the shift in context from extinction to the test phase.

Furthermore, the selective effect of extinction to an extinguished CS in Experiment 3 may suggest motivational mechanisms beyond inhibition. In appetitive motivation, Corbit and Balleine (2005) showed that presentation of Pavlovian stimuli associated with outcomes also earned by instrumental responses can have selective influence over choice between these responses. For example, a CS for food enhances responding on a lever that also earns food, while a CS for sucrose does the same but for a sucrose-reinforced response instead. Presentation of a Pavlovian CS predicting an outcome without an associated response available, by contrast, elevates responding non-specifically. These distinct behavioral effects are understood as reflecting sensory-specific and general motivation respectively, with further studies identifying unique brain circuitry involved in the different forms of motivation (Shifflet and Balleine, 2010; see Cartoni et al., 2016). In Experiment 3 we paired one CS (e.g., tone) with footshock and a different CS (e.g., noise) with klaxon, we then presented these stimuli over footshock avoidance. Based on the understanding of different forms of conditioned motivation from appetitive studies, a footshock-paired CS might be expected to facilitate footshock avoidance based on sensory-specificity to a greater extent than would a klaxon-paired CS. This cue would be expected to enhance responding on the basis of general motivation since no available response is associated with the klaxon. Thus while transfer was comparable as a function of outcome, and directed to the same response, the cues may have enhanced USAA differently.

Studies of aversive motivation have been limited in their ability to isolate these processes. In the typical aversive PIT study, a shock-paired CS enhancing footshock avoidance might be understood in terms of sensory specific motivation. However, studies in appetitive learning using only a single response do not seem to engage sensory-specificity in ways that designs involving richer associative experiences do (i.e., multiple CS-US associations or action-outcome contingencies; Holland and Gallagher, 2003). Therefore, it is difficult to determine whether the single response, single CS aversive PIT design generates transfer on the basis of sensory-specific or general motivation. However, because extinguished subjects in the within-subjects procedure did not generalize extinction from one CS to the other, this suggests that these cues enhanced USAA on the basis of distinct processes unique to each stimulus. With one being influenced by extinction, and the other not. Despite the limitations of this study (e.g., no inclusion of choice), the selective effect of extinction provides some evidence for distinct forms of aversive motivation, but not inhibition.

While the aversive PIT phenomenon utilizes avoidance behavior, it is primarily a phenomenon of expression or performance. For example, during signaled avoidance freezing to a tone that predicts shock is initially quite high, but over trials, Pavlovian extinction and negative reinforcement of target behaviors interact to shape the AR (see Choi et al., 2010; Moscarello and LeDoux, 2013; LeDoux et al., 2016). The same is true in unsignaled avoidance or USAA (see Lázaro-Muñoz et al., 2010; Martinez et al., 2013) where freezing to the avoidance context is initially high, and extinguishes over days as the AR emerges. This control over freezing has been shown to depend on prefrontal regulation of central amygdala (CeA) activity in a manner similar to what is known about how Pavlovian conditioned freezing responses alone are attenuated by extinction (Quirk et al., 2000; Moscarello and LeDoux, 2013). Whether a similar mechanism is involved in suppressing CS-elicited freezing during aversive PIT is not clear. However, the contribution of extinction processes to acquiring avoidance is distinct from the reduction in PIT-related motivation resulting from extinction treatment in the studies above. Reductions in Pavlovian reactions to conditioned stimuli appear a requisite for observation of consistent avoidance behavior and can even release latent avoidance learning when accomplished via lesions of the CeA in so called “poorly performing” subjects (see Choi et al., 2010). If expression of PIT were dependent on similar psychological mechanisms, one would expect extinction to further suppress CS-elicited freezing and increase the transfer effect even more so. This was clearly not the case: when extinction took place just prior to tests, it prevented PIT instead. These results suggest that the underlying motivation for integration of Pavlovian and response-contingent avoidance behavior in PIT may be different than for avoidance behavior alone. More work is needed to better understand this unique form of aversive motivation.

In summary, contrasting with studies of appetitive PIT, extinction was found to reduce the transfer of aversive motivation assessed via USAA rate when conducted prior to testing. This effect was found to be a selective reduction in transfer to the extinguished cue and not an enhancement in PIT for the non-extinguished cue as has been seen in appetitive motivation (Laurent et al., 2016). This suggests that distinct forms of motivation may underlie augmentation of USAA by stimuli predicting congruent as opposed to incongruent outcomes in a manner that is similar to findings in appetitive motivation.

## Author Contributions

VDC designed and ran studies, processed and analyzed data and wrote the manuscript. GR and ITK ran studies and processed data. ITK and JEL helped to write the manuscript.

## Conflict of Interest Statement

The authors declare that the research was conducted in the absence of any commercial or financial relationships that could be construed as a potential conflict of interest.
